# The Use of Carrot Fiber and Some Gums in the Production of Block-Type Melting Cheese

**DOI:** 10.3390/foods13132035

**Published:** 2024-06-27

**Authors:** Hasan Cankurt, Mustafa Cavus, Osman Guner

**Affiliations:** 1 Food Technology Department, Safiye Cikrikcioglu Vocational School, Kayseri University, Kayseri 38100, Türkiye; mustafacavus@kayseri.edu.tr; 2 Cookery Department, Develi Huseyin Sahin Vocational School, Kayseri University, Kayseri 38100, Türkiye; osmanguner@kayseri.edu.tr

**Keywords:** block-type melting cheese, carrot fiber, gum

## Abstract

In this study, the effect of carrot fiber and certain gums on the physicochemical, textural, microbiological, and sensory properties of block-type melting cheese, which holds a significant place in our daily food consumption, was investigated. The study also aimed to determine the impact of carrot fiber and other gums on cheese properties, as well as on yield and meltability. Carrot fiber was used at levels of 2.5% and 5.0% by weight, while carrageenan and xanthan gum were each used at levels of 0.25% and 0.50%. The cheeses were analyzed on days 1, 15, and 30. At the end of the study, it was determined that the highest total dry matter, fat, and protein values were found in the control sample due to the addition of water when preparing the cheeses with fiber and gum. The highest dry matter, fat, salt, and protein ratios were 59.65%, 29.40%, 1.48%, and 24.48%, respectively, in the control sample. The lowest fat, salt, and protein ratios were 25.00%, 1.31%, and 22.07%, respectively, in the 5.0% carrot fiber sample. The lowest dry matter value was found in the 0.5% xanthan sample, namely 53.62%. The highest L* value was measured in the control sample at 86.89, while the lowest was measured in the 5.0% carrot fiber sample at 81.86. The lowest a* and b* values were 2.82 and 29.42, respectively, in the control sample, while the highest values were 6.20 and 37.37, respectively, in the 5.0% carrot fiber sample. It was observed that the use of carrot fiber imparted an orangish color to the cheese. It was observed that the pH values of the samples were similar. According to the sensory evaluation results, the most liked sample was the control sample with 8.5 points, followed by the 0.25% xanthan sample with 8.0 points. The 5.0% carrot fiber sample received the lowest sensory appreciation with 6.1 points. It was understood that the use of carrot fiber gave the cheese an orangish color. Although the meltability varied according to the amount of gum and fiber used, it was measured at 6.92 cm in the 0.25% carrageenan sample on the first day and at 6.79 cm in the control sample on the last day of storage. It was observed that the use of fiber decreased the total bacterial count, while the use of gum increased it.

## 1. Introduction

Animal foods hold an important place among nutrition sources, with dairy products being particularly significant within this category. To meet the demand for these products, sufficient production is essential. Consequently, countries have recently turned to breeding milk-producing animals to fulfill this need [1]. In Turkey, cheese production has increased exponentially in recent years, driven by reduced costs resulting from advancements in production technology and the economic advantages it provides. Scientific studies on cheese have focused on the raw materials used in block-type melting cheese and the application of melting salts [2].

Cheese contains nutritionally important components such as calcium, protein, potassium, vitamin A, vitamin B12, and iron. The high-quality protein in cheese forms the building blocks necessary for the body to develop strong muscles. It has been reported that gums can be effective in reducing disorders such as cholesterol and diabetes that occur at certain stages of life. Recent scientific studies on gums have determined that they support live microorganisms that promote intestinal health [3]. 

Xanthan gum is used as an emulsifier in foods. It is an auxiliary substance suitable for food production because it does not adversely affect the taste of foods [4]. 

Gums used in the food production industry can be produced from three different sources: microbial, algal, and plant. They are widely used to give consistency to the product or for gelling. These are polymeric substances with the ability to dissolve in water [1].

Carrageenan is obtained from red seaweeds. The fluidity and flexibility of carrageenan gels can vary with temperature [5]. Xanthan gum is a polymer produced by certain living organisms. It was discovered in 1950 in a research laboratory in the United States [6,7].

Carrot (*Daucus carota* L.) is a food rich in carotenoids and dietary fibers, grown in large quantities worldwide [8]. Carrots hold a significant place in terms of human health. The regular consumption of fibrous foods like carrots can reduce the risk of cardiovascular diseases, diabetes, and digestive system issues. Carrots are commonly consumed in meals, salads, and even cakes. Besides their culinary uses, carrots contribute to healthy skin formation due to their antioxidant vitamin A content. Moreover, carrots contain essential minerals, vitamins, and enzymes that aid in blood cleansing and maintaining digestive system health. It is evident that both cheese and carrots are vital for human health.

Dietary fibers possess numerous technological functional properties. These include fat holding capacity, hydration properties such as swelling and solubility, water binding capacity, water retention capacity, and texture-improving properties [9]. 

Numerous studies have been conducted on the utilization of dietary fiber, which holds high health significance, in the production of milk and dairy products with an enriched fiber content [10]. On the other hand, it has been reported that dairy products, which may lack natural antioxidants and dietary fiber, can become nutritious alternative food products rich in health with the addition of dietary fiber [11]. 

In this study, the aim was to utilize carrot fiber, known for its numerous benefits mentioned above, in block-type melting cheese and compare it with other gums commonly used in this type of cheese. To achieve this, various ratios of gums and carrot fibers were incorporated into block-type melting cheese, and the cheeses were then stored for one month under cold and vacuum-packaged conditions. Analyses were conducted on the first day, in the middle, and at the end of the storage period to assess the physicochemical, textural, microbiological, and sensory properties of the cheeses.

Some gums are used extensively in the dairy sector, such as in ice cream and block-type melting cheese. However, the use of carrot fiber is not very common. The studies are still at the level of scientific studies and have not been reflected in the field. For this reason, it was aimed to investigate the effect of carrot fiber on block-type melting cheese and especially on color, hardness, sensory values, and yield. The other aim was to compare the performance of carrot fiber with the performance of carrageenan and xanthan gum, which are commonly used in this cheese. Fibers are known to contribute to intestinal health. For this reason, it was aimed to enrich the block-type melting cheese with carrot fiber, but since the health effects of this are the subject of other research, no analysis was carried out. 

## 2. Materials and Methods

### 2.1. Materials

During the execution phase of this study, whole cow’s milk obtained from a farm operating in Sakaltutan, Village was used in the production of block-type melting cheese (Talas, Kayseri, Türkiye). The properties of the raw milk used are provided in Table 1 below. Carrots were procured from the Metropolitan Municipality Fresh Fruit and Vegetable Market Complex, Kayseri, Türkiye. Gums were sourced from the Kayseri market. Some stages of cheese production were conducted at the Öz-Sütka company, a company operating in Kayseri, Türkiye, while others were carried out at the Kayseri University Safiye Cıkrıkcıoglu Food Studies Application and Research Centre. Analyses were also performed at this center. Properties of raw milk used in the production of melted cheese are given in Table 1.

### 2.2. Methods

The production methods of carrot fiber and cheese are given below. In addition, the methods of analyses are also explained.

#### 2.2.1. Carrot Fiber Production Method

In this study, carrots were first washed, and the stems were separated for carrot fiber production. Carrots were separated from the juice with a kitchen-type solid fruit juicer (Arnica nectarin plus), and the pulp remained. The pulp was pressed, and the remaining water was removed. Carrot pulp was dried at 50 °C for 6 h. The dried carrot pulp was ground and pulverized with a coffee and spice grinding machine (Kiwi brand). The obtained carrot fiber was passed through a flour sieve, and carrot fiber was obtained. The obtained carrot fibers were stored in glass jars in a humidity-free and light-free environment.

#### 2.2.2. Block-Type Melting Cheese Production Method

**Figure 1:**
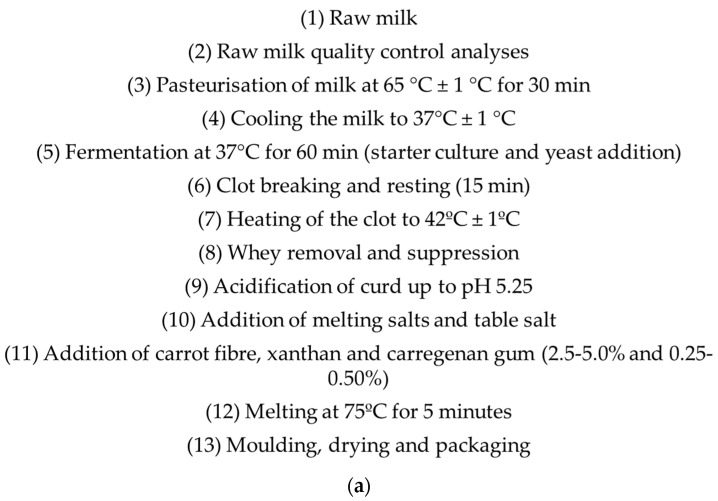

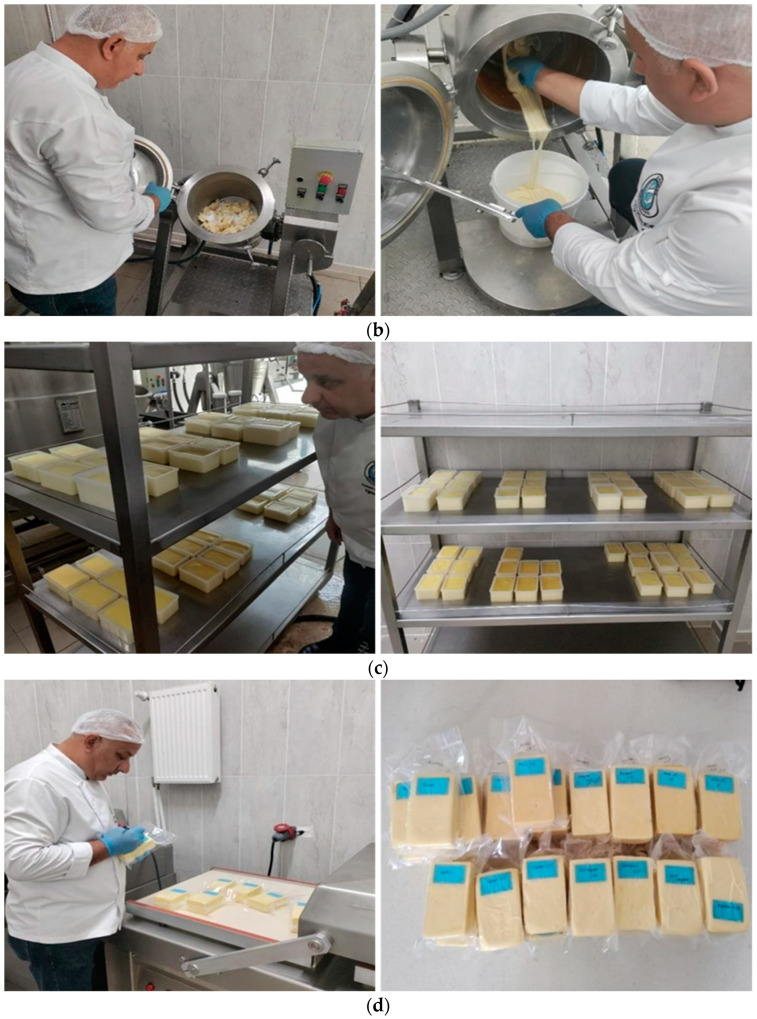
Some Images from the Production Stages of Melted Cheese. (**a**) Block-Type Melting Cheese Production Flow Chart. (**b**) Putting the cheese curd into the machine and removing the melted dough from the machine during the preparation of cheese samples. (**c**) Molding the obtained cheeses and keeping them in the mold. (**d**) Packaging of the cheeses.

Below, the production stages of block-type melting cheeses are given in the form of a flow chart. Figure 1. shows a few images of the production, drying, and packaging stages.

The yield of cheese was found by calculation. Yield results are given as the amount of cheese obtained from 100 L of milk [11]. The amounts of cheese obtained from each 100 L of milk are given below. Since the non-control cheeses contained gum or fiber, it was necessary to add water to the cheese dough. Therefore, the yield of non-control cheeses was higher. The amounts of cheeses obtained are given in Table 2.

#### 2.2.3. Physicochemical Analyses

After adding 40 mL of distilled water to 10 g of cheese sample and homogenizing with an Ultra Turrax homogenizer (Wise Tis HG-15D), the pH of the mixture was measured using a table pH meter (Hanna-Instrument) [12]. 

A total of 10 g of the cheese sample was weighed and homogenized in an ultraturax device by adding 40 mL of distilled water. The homogenized cheese sample was titrated with 2–3 drops of 1% phenolphthalein and 0.1 N NaOH until a permanent light pink color was obtained. The result was calculated using Equation (1) and expressed as % total acidity [13].

% Total Acidity = (V × 0.009 × 100)/m (1)

V: Volume of 0.1 N NaOH consumed in the titration (mL)

m: The amount of sample (10 g) used in the titration.

The gravimetric method was used to determine the dry matter content in cheese. Drying containers were taken into the oven and subjected to a drying process. After cooling in the desiccator, they were tared, and then 3 g of the cheese sample was weighed and crushed into small pieces. It was kept in the oven at 105 °C for 4 h until it reached constant weight. After reaching constant weight, the sample was removed from the oven and cooled in a desiccator. The cooled sample was calculated according to Equation (2), and the result was expressed as % dry matter [13].

Dry matter (%) = (m1 − m)/(m2 − m) × 100 (2)

m: Drying container tare (g)

m2: Weight of the sample and drying container before drying (g)

m1: Weight of the sample and drying container after drying (g)

The ash content of cheese samples was determined by incineration in an ash furnace (Elektromag, İstanbul, Türkiye). The pre-cleaned crucibles were dried to constant weight, and the tare was recorded. Then, 3 g of the sample was weighed in the crucible. The crucibles were kept in an oven at 110 °C for 4 h to dry. Then, they were placed in the muffle furnace at 520 °C and kept there for 1 night. The incineration process was terminated when constant weight was reached. Then, the crucibles were placed in the desiccator and kept there until they reached room temperature, and the total weight of the ash and crucible was recorded. The results are calculated according to Equation (3) [14].

% Ash = [(M2 − M1)/m] × 100(3)

M2 = Crucible after incineration + ash weight

M1 = Weight of the crucible brought to constant weight

m = Weight of sample taken 

The fat content of the cheeses was determined by applying the Gerber method. A total of 3 g of crushed cheese was weighed into the beaker of the cheese butyrometer. Sulfuric acid (H_2_SO_4_) with a density of 10 mL 1.55 g/mL (H_2_SO_4_) was added to the cheese and stirred at intervals in a 65 °C water bath until the cheese was completely dissolved. Then, 1 mL of amyl alcohol was added. The rest was completed with sulphuric acid (H_2_SO_4_), and the stopper was tightly closed. After centrifugation for 5 min, it was kept in a 70 °C water bath for 5 min, and the oil content was determined as % using a butyrometer scale [15].

The fat content in dry matter was determined by dividing the fat content of the cheese by the dry matter content and multiplying by 100. 

The salt content of the cheese samples was determined by titration with 0.1 N silver nitrate using the Mohr method and expressed as % salt content. For this reason, 5 g of the samples were taken and thoroughly crushed, and then 65 °C hot water was added to 500 mL and the salt allowed to pass into the water. The mixture was filtered through coarse filter paper, and 25 mL of this filtrate was taken and titrated with 0.1 N silver nitrate after potassium chromate was added as indicator. The amount of salt was calculated according to Equation (4).

(4) $$\%\;\mathrm{Salt}\;\mathrm{amount}\;(g/100\;g)=\frac{\left(V2-V1\right)\times N\times mEq\times F\times 100}{Sample\;weight\;\left(g\right)}$$

V1 = Amount of AgNO_3_ to be used in the witness trial (mL)

V2 = Amount of AgNO_3_ to be used in the main trial (mL)

N = Normality of AgNO_3_ solution

mEq = milli-equivalent weight of NaCl (0.0585 g)

F = the factor of the AgNO_3_ solution.

Protein ratios were calculated by multiplying the nitrogen content of the samples subjected to wet digestion determined by the Micro Kjeldahl method by the multiplication factor of 6.38 applicable to dairy products and expressed as %. Protein content in total dry matter was calculated according to Equation (5) [16].

(5) $$\%\;\mathrm{Protein}\;\mathrm{at}\;\mathrm{total}\;\mathrm{dry}\;\mathrm{matter}=\frac{\%\;protein\times 100}{\%\;total\;dry\;matter}$$

#### 2.2.4. Color Analysis

A color analyzer (Hunterlab, Reston, VA, USA) was used to analyze the color properties of block-type melting cheeses. L*, a*, and b* color values were measured. 

#### 2.2.5. Fusibility

This analysis was carried out using a modified Schreiber test. Cheese samples at 4–6 °C were cut into circles of 40 mm in diameter and 5 mm in height and placed in the centre of a petri dish. The petri dish was then placed in an oven at 230 °C for 5 min. After removal from the oven, the petri dishes were placed on a flat surface and left for 30 min to cool down at room temperature. At the end of the time, the spreading diameter in the petri dish was measured from six different points, and the amount of spreading was determined by averaging the spreading diameter measurements made at different points [17].

#### 2.2.6. Microbiological Analyses

After serial dilutions of the samples were prepared, these dilutions were incubated for 24–48 h at 30 °C using the Plate Caunt Agar (PCA, Merck) surface spreading method, and the result was determined by counting the colonies that developed at the end of incubation [18].

After serial dilutions of the samples were prepared, these dilutions were inoculated onto VRB Agar (Merck, Darmstadt, Germany) by the pouring method and incubated at 37 °C for 24 h. The colonies were counted and counted as coliform bacteria [18]. After serial dilutions of the samples were prepared, these dilutions were inoculated onto Dichloran Rose Bengal Cloramphenicol Agar (DRBC) (Merck) using the surface spreading method, and the petri dishes were incubated at 25 °C for 3–5 days. Typical colonies judged to be yeast and mold were counted [19].

#### 2.2.7. Textural Analyses Applied to Cheeses

According to the method described by Cankurt [20], textural properties of the cheese samples were determined using a TA.XT Plus Texture Analyzer texture-measuring device. The samples to be analyzed were cut into 4.0 cm cubes. A spherical head with a diameter of 4 cm was used for pressing. Using a 30 kg load cell, the compression speed was set as 1 mm/s, and the total process time was set as 10 s. The compression process was carried out to compress 25% of the original size of the samples. According to the tissue profile analysis technique, the tissue profile of the samples was determined by measuring the tissue profile analysis parameters, which are hardness (kg), internal cohesiveness (cm^2^), elasticity, external cohesiveness (cm^2^), chewiness (kg), and gumminess (kg). 

#### 2.2.8. Sensory Analyses

Sensory analyses of the cheeses were carried out by expert panelists according to the scoring method. Cheese samples were prepared in 15–20 g portions after being taken out of cold storage. The panelists were first trained and informed about the scoring of the cheeses according to the sensory quality criteria. The cheeses were coded and presented to the panelists. The coding used was: 126 (0.25% carrageenan gum-added cheese sample), 318 (0.50% carrageenan gum-added cheese sample), 254 (0.25% xanthan gum-added cheese sample), 489 (0.50% xanthan gum-added cheese sample), 771 (5% carrot fiber-added cheese sample), 605 (2.5% carrot fiber-added cheese sample), 167 (Control group cheese sample). The samples were given to the panelists together with bread and water. The panelists were asked to evaluate the cheeses in terms of appearance, texture, flavor, and overall impression quality characteristics and to indicate the defects that caused a decrease in the score [21].

### 2.3. Statistical Analyses 

In this study, the microbiological, physicochemical, textural, and sensory properties of the melted cheeses produced and stored in this study were studied with the JMP PRO package programme, Version 27 (SPSS Inc., Chicago, IL, USA) and recorded by one way ANOVA. Within the scope of the study, seven different cheese samples (control group, carrot fiber, xanthan gum-, and carrageenan gum-added cheeses) were statistically analyzed in three different storage periods (1st day, 15th day, 30th day) with three replicates. As a result of the analyses, significant differences between the averages were determined by the Tukey multiple comparison test at *p* < 0.05 significance level [22].

## 3. Results and Discussion

In this section, the findings obtained throughout the study are presented and discussed in the context of the literature. The reasons for some of the significant results obtained are outlined. Each topic is discussed in its respective section. The findings are initially presented in a table format, followed by a discussion of the results in relation to the existing literature.

### 3.1. Physicochemical, Color and Fusibility Results of Block-Type Melted Cheeses

In this study, fat and dry matter fat values, dry matter, salt and dry matter salt values, pH value, total acidity value, protein values, and color values (L*, a*, b*) of block-type melting cheeses were measured. 

In our study, only the first-day analyses were performed on these data considering the previous studies, since the fat, fat in dry matter, protein, dry matter, salt, salt in dry matter, and ash ratios of the cheese samples stored in vacuumed and packaged form would not have changed during storage. Dry matter values of block-type melted cheese samples are given in Table 3, fat values are given in Table 4, fat in dry matter values are given in Table 5, salt values are given in Table 6, salt values in dry matter are given in Table 7, protein values are given in Table 8, ash values are given in Table 9, pH values are given in Table 10, total acidity values are given in Table 11, L* values are given in Table 12, a* values are given in Table 13, b* values are given in Table 14 and fusibility values are given in Table 15.

When the analyses from the first day were examined, it was observed that the salt content of the cheese samples ranged between 1.31% and 1.48%. These results are detailed in Table 6. In previous studies, Çavuş [23] determined the salt content of the control cheese samples as 1.59% in the first-day analyses. Other researchers determined the salt content of control cheese samples as 3.26% in Öztek [24], 3.16% in Ayar [25], 1.78% in Yüksel [26], 1.91% in Koca and Metin [27], 1.17% in Keçeli et al. [28], and 4.09% in Ayar et al. [29]. Since there may be changes in salt ratios due to the demands of consumers and the production methods of the producer, it was thought that the comparison would not be healthy. Some of the findings given by previous researchers are the salt content of kashkaval cheese. After the kashkaval cheese is salted in salted boiling water, salt is added during kneading. In this way, the shelf life is extended, because it is a cheese that is consumed by ripening. However, less salt is used in fresh block-type melting cheese. For example, Cankurt [20] measured the effect of different hydrosols on some properties of block-type melting cheese and found salt ratios between 2.04–2.06%.

Salt content in the dry matter of the cheese samples was determined between 2.43% and 2.51%. In the study conducted by Çavuş [23] on the production of block-type melting cheese, it was determined that the amount of salt in dry matter was between 2.87% and 2.98%. In the study conducted by Yüksel [26] on the production of dietary block-type melting cheese, the amount of salt in dry matter was determined as 3.83% and 4.08%. Cankurt [20] found salt ratios in total dry matter between 3.44% and 3.62% in his study on hydrosol block-type melting cheese. The salt and dry matter salt ratios in block-type melting cheese vary according to the formula of the researcher. Therefore, comparing the results gives an idea but does not make a clear judgement. 

The ash ratios of the block-type melting cheeses produced were determined as 3.52% at the lowest and 3.97% at the highest. The values are shown in Table 9. As a result of the ash analysis of kashkaval cheese performed by Tavacı [30], ash ratio was determined between 4.31–4.53%. In different studies conducted on kashkaval cheese, Fırat [31], Ayar [25], Demirci and Dıraman [32], Kurultay [33], and Özdemir [34] reported ash ratios between 3.05–5.78%. A large part of the ash value is due to salt. The higher the salt content in a cheese, the higher the ash value. In addition, herbal flavorings added to cheeses are also among the factors affecting the ash value. In addition, if the added salt contains other minerals in excess, it causes an increase in the ash ratio to the same degree [20]. Since the salt content is relatively low in our cheeses, the ash content is also low. It is seen that the difference in the ash ratios of the cheese samples is mainly due to the dry matter content, because the highest ash content was measured in the control sample with the highest dry matter content.

The lowest dry matter values of the block-type melting cheese samples were found to be 53.62%, and the highest 59.65%. Ayar [25] analyzed cheese samples produced in Trabzon market and found that the average dry matter content was 57.34%. In a study conducted by Coşkun and Öztürk [35] on cheeses produced in Van and offered for consumption, it was found that the lowest was 44.02% and the highest was 63.54%. When the analyses of other studies were examined, it was found that there were differences. Keçeli et al. [28] found an average of 46.56%, while Estürk [36] found this value to be 56.37%. The results obtained in our study were found to be closer to the results obtained by Estürk [36]. Block-type melting cheese can be produced at desired dry matter values. In this cheese, the texture is provided by the product content, and emulsifying salts are used as a support. Some of them provide hardness, while others provide hot meltability and creep. Therefore, the dry matter content of cheeses depends entirely on the will of the producer [20]. In our study, the dry matter of all samples containing gum and fiber was found to be lower than the control sample, because gums have water binding properties. In our study, less additional water was added to the samples containing low levels of gum and fiber, while more water was added to the samples containing high levels of gum and fiber, so the dry matter values varied. 

The fat values obtained from the block-type melting cheese samples we produced ranged between the lowest at 25.00% and the highest at 29.40%. Detailed fat values are provided in Table 4. In the study conducted by Demirci and Dıraman [32], the fat content of fresh kashar cheese was found to be 24.11%. In a study conducted by Ayar [25] in which the properties of cheeses produced in Trabzon were investigated, it was reported that the fat ratio was found to be 25.10%. In another study conducted by Fırat [31], the fat content was determined as 27.66%. Cankurt [20] investigated the effect of some plant hydrosols and essential oils on the physicochemical, textural, microbiological, and sensory properties of block-type melting cheese and found fat ratios between 29.77% and 31.50%. The fat content in cheese is affected by the fat content of the raw milk used, the heat treatment applied to the milk, and other production stages, as well as the ratio and type of other ingredients added. The milk used in our study is the same. However, although the rate of gums added to the formulations is low, the rate of carrot fiber added is high enough to affect the overall composition. Accordingly, it was expected that the control sample, which had the highest dry matter content, would have a high ratio of gum. In the carrot fiber 5.0 sample, the oil ratio was low because the amount of water and carrot fiber used was high. 

Fat ratios in dry matter were determined as 46.35% at the lowest and 49.52% at the highest. When the studies on kashkaval cheese were analyzed, it was found that the fat ratios in dry matter were 40.97% by Fırat [31], 42.79% by Öztek [24], 43.63% by Ayar [29], 38.6% by Estürk [36], and 32.77% by Keçeli et al. [28]. Cankurt [20] found the oil content in total dry matter to be between 52.41% and 53.24%. The results found in our study are between the results of this researcher and the results of Öztek [24]. The fact that other researchers found lower results is due to the fact that either they used less fat milk or some of the fat passed into the water while boiling in water. The lower fat content in the total dry matter in the carrot fiber 5.0 sample is due to the fact that 5% fiber was included in the composition. 

In the pH analyses of block-type melting cheeses, a decreasing trend was observed from the first day to the last day. On the first day, pH values varied between 5.63 and 5.70. In the 15th day analyses, pH values ranged between 5.65 and 5.70, and in the 30th day analyses, they varied between 5.58 and 5.66. The pH values are presented in Table 10. In other studies, Keçeli et al. [28], Demirci and Dıraman [32], and Estürk [36] reported pH values between 5.13 and 5.70. Say [37] reported that the lowest value was 5.30 on the first day and 5.17 on the last day of storage in a study on the properties of cheese during storage. Tavacı [30] determined the pH value of the cheeses he produced as 5.42–5.47, and Fındık [38] determined that the pH value was between 5.27 and 5.29 in the production of fresh kashkaval cheese. In our study, it was observed that our pH values were higher than those of other researchers. Cankurt [20] found the pH values of the samples to be between 5.73–5.91 on the 30th day of storage. Our results appear similar to those of this researcher, because when we look at the production methods, it can be said that they are exactly the same. The reason why other researchers’ cheeses have low values is that they are generally produced by boiling with water. In such a case, the internal temperature remains around 57 °C. As a result, lactic acid bacteria remain largely alive, lactic acid development continues, and the pH value decreases. However, melted cheese is boiled in a Stefan type machine and usually at 75 °C. Additionally, pH-increasing emulsifying salts are added to the structure. For this reason, the pH value of melted cheese is higher. Melting salts are not used in wet boiled kashar cheese [20]. In our study, it was understood that the pH values were not different enough to be discussed. Accordingly, the use and amounts of gum or carrot fiber did not significantly affect the pH value.

When examining the total acidity values of the block-type melted cheese samples, it was observed that the acidity increased in all samples. On the first day analyses, it ranged between 1.45% and 1.72%. In the 15th day analyses, it varied between 1.55% and 1.77%, and in the 30th day analyses, it ranged between 1.54% and 1.83%. Özdemir [34] and Çağlar and Çakmakçı [39] reported that the acidity ratios of kashkaval cheese samples increased continuously during storage. Öksüztepe et al. [40] reported the average titration acidity value as 0.42% in their study on the microbiological and chemical qualities of kashkaval cheese produced in Elazığ. Aydın [41] reported that titration acidity values in kashkaval cheese samples were between 1.17 and 1.66%. Yaşar [42] found the titration acidity values determined during the ripening period of kashkaval cheese as 0.70% and 2.15%, respectively. Cankurt [20] determined total acidity values as 1.47 to 1.84%. In our study, total acidity values were found to be similar to those found by Cankurt [20]. While the results of some researchers were found to be close to our results, some were found to be significantly lower. The reason why it is low is that kashar cheese is boiled in water. Accordingly, the lactic acid formed in the curd passes into the boiling water during boiling. Thus, the total acidity is weak. However, the live lactic acid bacteria in these cheeses increase the total acidity again over time. As a result, total acidity varies depending on many factors such as the level of heat treatment applied to the milk, boiling method, starter culture addition, storage temperature, packaging type, and the amount of water in the composition. Since there was no wet boiling in our study, the acid formed in the curd remained in the resulting cheese. Therefore, the acidity on the first day was relatively high. This acidity increased with storage, albeit slowly. In this study, no significant relationship was found between increasing the water amount and increasing the gum and total acidity. 

When examining the protein values of block-type melted cheeses, the lowest protein value was determined to be 22.07% in the cheese sample with 5% carrot fiber, while the highest value was determined to be 24.48% in the control group cheese sample, as shown in Table 8. Cankurt [20] reported that the protein values of cheeses were between 25.71 and 27.48%. Çavuş [23] stated that the protein values of the cheeses he produced were between 26.55 and 28.75%. Aydın [41] found protein values between 20.17% and 25.01% in the study of the effects of different herb varieties used in cheese production on ripening. Hayaloğlu [43] stated the protein values of cheeses as 12.78% and 17.21% in his study on the properties and ripening of white cheeses. Bayram [44] reported that the protein values of the cheeses produced were between 23.17 and 25.83%. When similar studies are analyzed, it is thought that the reasons for the occurrence of different protein values may be due to the method of cheese production, the type of animal from which the milk used comes, nutritional status, and the amount of salt added to the cheese. Cankurt [20] reported that total protein in block-type processed cheese decreased by around 0.7% after 90 days of storage. However, we only measured the total protein ratio in the first day analysis, anticipating that there would be no decrease. The difference in the protein values of our samples is largely due to formulation differences. The fact that some researchers’ protein ratios are lower than ours is most likely due to production differences, because in the wet boiling method, not only is fat lost, but water-soluble proteins and proteins combined with fat are also removed. 

When examining the L* values of the color values of the block-type melting cheese samples produced, differences were observed. On the first day analyses, L* values varied between 82.02 and 87.41. On the 15th day analyses, they ranged from 79.86 to 88.09, and on the 30th day analyses, they ranged from 81.86 to 87.81. The highest L* value was 88.09 in the cheese sample in which 0.50% carrageenan gum was used, while the lowest L* value of 79.86 was found in the cheese sample with 5% carrot fiber added. In the study by Fırat [31] titled “Determination of some microbiological, physical and chemical properties of kashkaval cheese during ripening”, the highest L* value was 87.30 in control kashkaval cheese samples. Cankurt [20] found the highest L* value as 90.38 in control cheese samples. The L* values obtained in our study are similar to the L* values obtained by other researchers. Our samples were found to be different within themselves. While the use of carrageenan whitened the color, the use of carrot fiber reduced the whiteness. While some of the components in cheese have a reducing effect on whiteness, components such as calcium increase whiteness. Since the water content is high in those with high gum content, the components that suppress whiteness decreased proportionally, and thus the whiteness of the samples containing carrageenan and xanthan gum with high water content was found to be higher. However, this was not the case in samples containing carrot fiber. While carrot fiber is produced, the fibers are not washed. Therefore, they contain significant levels of colorants. Since carrot fiber has a reducing effect on whiteness, whiteness decreased as carrot fiber increased. Çavuş [23] reported that the L* values of block-type melted cheeses produced using eggs decreased with storage, the highest values were in the control cheese, and the lowest values were in the sample with egg yolk added. Öksüz et al. [45] found the lowest L* value in the kashar cheeses they produced to be 74.8. They found the highest to be 90.1. The L* values we obtained are consistent with the findings of previous researchers.

Significant differences were observed when examining the a* values among the color values of cheese samples. On the first day analyses, a* values ranged between 2.92 and 5.49. In the 15th day analyses, they were observed to vary between 2.53 and 5.86, and in the 30th day analyses, between 2.82 and 6.20. The highest a* value was observed in the cheese sample to which 5% carrot fiber was added, with 6.20. The lowest a* value was found in the cheese sample with 0.50% xanthan gum added, at 2.53. Except for the carrot fiber cheese samples, the other cheese samples showed a decrease in the 15th day analyses compared to the first day analyses, but it was found to increase again in the 30th day analyses. Carrot fiber-added cheese samples showed an increase in the values starting from the first day analyses. In the study on the determination of some microbiological, physical, and chemical properties of kashkaval cheeses produced by Fırat [31] at the ripening stage, it was reported that a* values were between −4.50 and −4.54. Öksüz et al. [45] reported that the a* value was between −0.76 and −7.8 in the color analysis of kashkaval cheese. Cankurt [20] reported that the a* value was between 0.40 and 2.05 in his study. Aydın [41] stated that the a* value of cheese samples was between −3.46 and −0.90. The color values in cheese are, of course, affected by the composition of the final product, the cheese, and the raw milk used in the production of this cheese. However, components that are added later and have coloring properties also have a significant effect on color. It was observed that the carrot fiber cheeses obtained in our study were redder. Since the carrot fibers were dried without washing, they gave the cheese an orange color due to the beta carotene it contains. It has been observed that other gums used also have an effect on the a* value and that this effect becomes more evident over time.

When examining the b* value of the cheese samples, it ranged between 28.08 and 33.99 in the first day analyses. In the 15th day analyses, it varied between 29.00 and 34.70, and in the 30th day analyses, between 29.42 and 37.37. The highest b* value was 37.37 in our cheese sample with 5% carrot fiber added, while the lowest b* value was 28.08 in the cheese sample with 0.50% carrageenan gum added. It was observed to increase from the first day to the last day of storage. Say [37] reported that the b* value varied between 28.03 and 36.33 during the storage process in the study on the effects on the properties of kashkaval cheese. In different studies, the b* value was determined by Öksüz et al. [45] at 19.24–28.87, Cankurt [20] 19.30–24.19, Göncü [46] 22.38–30.33, and Çavuş [23] 18.32–39.12. Beta carotene in carrots increases both redness and yellowness. For this reason, the b* value of the carrot fiber-added samples was found to be higher. Accordingly, it was observed that cheeses with increased yellowness and redness were sensory orange, as expected. The use of other gums, mostly in carrot fiber samples, also increased the yellowness of the cheeses. 

The fusibility values of the block-type melting cheese samples are provided in Table 15. Upon an examination of the samples, differences were observed in the analyses conducted during storage. In the first day analyses, fusibility varied between 5.87 cm and 6.92 cm. In the 15th day analyses, it was observed to range between 5.79 cm and 7.04 cm, and in the 30th day analyses, between 5.92 cm and 6.79 cm. The highest fusibility value was 7.04 cm in the cheese sample with 0.25% carrageenan gum, while the lowest fusibility value was found in our cheese sample with 5% carrot fiber added, measuring 5.79 cm. When the fusibility data were examined, it was seen that all gums and carrot fiber reduced the fusibility to an acceptable level. In kashar and block-type processed cheeses, fusibility is mostly associated with protein content, calcium content, and pH value [20]. These features of our cheeses are close to each other. However, carrot fiber samples have a lower protein content. In fact, the fusibility of carrot fiber samples was found to be lower. However, it can be said that the use of gum and carrot fiber physically prevents the fusibility of the cheese samples.

### 3.2. Microbiological Results of Block-Type Melted Cheeses 

In this section, first of all, the results are presented in tables. The results obtained were discussed in light of the literature. TAMB count of block-type melted cheese samples is given in Table 16, total yeast-mold count is given in Table 17, TLAB count is given in Table 18 and coliform group bacteria count is given in Table 19.

In our study, coliform bacteria were detected in some cheese samples on the first day of the storage period. However, no coliform bacteria were detected in subsequent analyses during storage. It is hypothesized that the reason for this is that the milk used in cheese production is pasteurized, and additionally, the scalding process is applied at 72 °C for 1 min during cheese production, which prevents the multiplication of coliform group bacteria and inhibits them from reaching a dangerous level. Tavacı [30] and Babacan [47] reported that kashkaval and melting cheese did not contain coliform bacteria. 

The yeast and mold counts of the cheese samples are shown in Table 17. As a result of the analyses, the amount of yeast and mould on the first day varied between 2.00 and 2.65 (log cfu/g). On the 15th day, it was observed to vary between 2.30 and 3.25 (log cfu/g) and on the 30th day between 2.39 and 3.35 (log cfu/g). The highest yeast-mold count was 3.35 (log cfu/g) in the cheese sample with 0.50% carrageenan gum. The lowest yeast-mold count was found in the control cheese sample with 2.00 (log cfu/g). Topal [48] reported that the lowest yeast-mold count was 3.48 log cfu/g and the highest was 9.92 log cfu/g in his study on the effect of storage on the surface mold and quality characteristics of cheddar cheese. Kıvanç [49] reported that the lowest yeast-mold count was 3.56 log cfu/g and the highest was 6.44 log cfu/g in a study on kashkaval cheeses produced and consumed in Erzurum province. Coşkun and Öztürk [35] found the lowest count to be 2.20 log cfu/g and the highest 5.98 log cfu/g. Kurultay [33] reported the average yeast-mold count as 5.88 log cfu/g. Kınık et al. [50] stated that yeasts and molds cause undesirable taste, flavor, and structure in cheeses. Cankurt [20] found the total mold-yeast count in the hydrosol and volatile oil block-type processed cheeses he produced to be between 2.24 and 4.24 log cfu/g on the first day and between 4.15 and 6.82 log cfu/g at the end of 90 days of storage. The total mold count of the samples is affected by many factors such as the hygiene of the environment where the cheese is produced, personnel hygiene, heat treatment applied, starter culture used, ripening environment, water content, and packaging method [20]. For this reason, the total mold-yeast load of the samples found by each researcher is different. In our study, the lowest mold-yeast count was detected in the control sample. Since the lowest humidity rate was also detected in the control sample, it is considered that the difference may be due to the humidity rate.

Total mesophilic aerobic bacteria counts in block-type melted cheese samples are given in Table 16. The lowest value was found in the cheese sample to which 2.5% carrot fiber was added with 2.87 log cfu/g. The highest value was found in the cheese sample to which 0.50% xanthan gum was added with 6.32 log cfu/g. Total aerobic mesophilic bacteria numbers are very interesting. The number was found to be very high in xanthan samples from the first day. In this case, it can be thought that the xanthan used was contaminated with bacteria. On the contrary, the number was found to be lower in carrot fiber samples. Although there was an increase in the number until the end of storage, the lowest number was still detected in these samples. Clearly, carrot fiber suppressed the total number of mesophilic aerobic bacteria in cheeses. The use of xanthan also promoted these bacteria. How this effect occurs in both directions and the reasons that cause it need to be investigated. 

The lowest total lactic acid bacteria count was 2.48 log cfu/g in the 30th day analysis in the cheese samples with 2.5% carrot fiber added. The highest value was observed in the 30th day analysis, with 6.90 log cfu/g in the cheese samples with 0.50% xanthan gum added. The same comments made for total mesophilic aerobic bacteria numbers can also be made for total lactic acid bacteria, because the results are exactly parallel. Accordingly, the effect of xanthan and carrot fibers was also confirmed on the total lactic acid bacteria counts. 

### 3.3. Results of Sensory Characteristics of Block-Type Melted Cheese 

During the storage period, sensory analyses, including of appearance, texture, flavor, and overall impression, were conducted on the first, 15th, and 30th days. The analyses were performed by 10 panelists aged between 25 and 55 years, who were experts in their fields, and they scored on a scale of 9 points. Prior to the analyses, the panelist group was informed and instructed to evaluate accordingly. 

Here, first the values are given in tables, and then the findings are discussed. Appearance values are given in Table 20, texture values are given in Table 21, taste values are given in Table 22 and all impression values are given in Table 23.

The appearance tests of the produced cheese samples were conducted by panelists. The appearance values of the cheeses ranged between 6.6 and 8.5 points. The highest score of 8.5 was awarded to the control group cheeses in the 30th day analyses, while the lowest score was given to the cheese samples with 5% carrot fiber added in the 30th day analyses. The cheese sample that consistently scored below 7 points throughout the analyses was the one with 5% carrot fiber added. It is believed that the reason it received fewer points than the other cheeses is that the addition of carrot fiber imparted a different color, which may have influenced the panelists’ perceptions. It was expected that samples with added carrot fiber would be appreciated more. However, carrot fiber gave the samples a slightly duller orange color. In addition, the visible size of the carrot fibers reduced appreciation. Since the additional water used in the samples with too much fiber and gum added to them gave these samples a wet appearance and a slight grayness, their appearance was less appreciated than the control sample. 

When evaluating the texture properties of the produced cheese samples, it was found that they received scores ranging between 5.5 and 8.6. The lowest score in texture values was obtained by the cheese sample with 0.50% xanthan added in the first day analyses. The highest score of 8.6 was achieved in the 30th day analysis, specifically in the control group cheese sample. It was expected that the samples using gum would feel softer due to the water added to the samples. However, the gums used bound the water. On the other hand, the texture of those with high amounts of gum was weakened, because the amount of water added was also high. Although they were produced by adding water to carrot fiber samples, they had similar values to the control cheese on the first day. They kept their scores until the end of storage. The fact that there was no sensory hardening or softening in the samples over time shows that these cheeses are more stable in terms of texture.

The cheese samples scored between 5.6 and 8.4 points in the flavor test. The lowest score of 5.6 points was recorded in the 15th and 30th day analyses for the cheese sample with 5% carrot fiber added, while the highest score was obtained by the control group cheese sample in the 30th day analysis. All impression values are shown in Table 23. The flavor of carrot fiber is not desired in block-type melted cheese. Perhaps cheeses would be more appreciated if the flavor of the fibers was neutralized. It was observed that the samples other than carrot fibers received quite high scores, although not as high as the control. Accordingly, it can be said that the use of gum does not significantly negatively affect taste in terms of sensory aspects. 

All impression tests of the cheeses received scores between 5.8 and 8.5. The lowest score of 5.8 was attributed to the cheese sample with 0.50% xanthan gum added in the first day analysis, while the highest score of 8.5 was awarded to the control group cheese sample in the 30th day analysis. It has been understood that the samples containing xanthan and carrageenan are generally appreciated and can be used successfully in block-type melted cheese. However, it was observed that the general appreciation was low due to the decrease in both appearance and taste caused by the addition of carrot fiber. In general, the general appreciation of cheeses other than carrot fiber samples increased due to the improvement in taste and texture as the cheeses matured.

### 3.4. Results of Textural Properties of Block-Type Melted Cheese 

Texture is an important factor in cheese production. Different parameters can be obtained in texture profile analyses [51]. Salt, dry matter, and pH affect the texture of the cheese produced [52]. When the texture parameters of cheese are analyzed, they are compatible with each other except internal stickiness. The fat in the dry matter is only associated with elasticity and hardness [53]. 

In this study, a Texture Profile Analysis investigated the effects of the addition of carrot fiber and some gums to cheese and ripening time on different parameters such as hardness, external stickiness, elasticity, internal stickiness, gumminess, and chewiness. The results are tabulated below. Afterwards, they are discussed in light of the literature. Hardness (g) values are given in Table 24, external stickiness values are given in Table 25, elasticity values are given in Table 26, internal stickiness values are given in Table 27, gumminess values are given in Table 28 and chewiness values are given in Table 29.

Şanlı [54] reported that although there are sometimes fluctuations in the hardness values of cheeses, it is generally in a decreasing trend. Another study on cheese [21] examined the use of fat substitutes in cheese and found that hardness values fluctuated but generally decreased. Another researcher reported that Camembert cheese was hard at the beginning of storage but softened later [55]. When we examined the hardness values of the cheese samples we produced, it was observed that the samples containing different additives were softer. Cheese samples in which no additives were added were found to be harder than the other samples. Cankurt [20] found hardness values of block-type melting cheeses produced by using different aromatic plant juices and essential oils between 1850–3052 g in samples containing aromatic plant juice and 2141–4813 g in samples with essential oil. He attributed the lower hardness values of the samples with hydrosol to the lower dry matter of these samples. Yüksel [26] found hardness values between 1874–2752 g in his study in which he used eggs in block-type melting cheese production. In our study, since the gum and fiber ratio was increased in the xanthan and carrot fiber samples, the water ratio was also increased, and therefore the hardness decreased significantly. However, in the carrageenan samples, the increased amount of carrageenan prevented the added water from softening the cheeses. The hardness values of our samples varied between 1189–4035 on the first day. These values are similar to the values found by Cankurt [20] and Yüksel [26]. In the sample to which carrot fiber was added, it was observed that carrot fiber could not retain the added water because the water retention capacity of carrot fiber was lower than that of carrageenan. 

Antoniou et al. [56] stated that the external stickiness of cheese is called the negative force field at the time of initial compression. Another researcher referred to the force applied to separate the cheese adhering to the palate with the help of the tongue as external stickiness [57]. The external stickiness values of block-type melting cheese were analyzed. As a result of the analyses, it was determined that the external stickiness values of the cheeses produced were between −24 and −760. The cheese sample with the highest external stickiness value was found in the first day analysis of the cheese sample to which 0.50% carrageenan gum was added. The lowest value was −24 in the 30th day analysis of the cheese sample with 0.50% xanthan gum. The values are shown in detail in Table 25. Cankurt [20] reported that external stickiness values were found between −2.58 and −8.55 on the first day and between −19.13 and −53.02 at the end of 90 days of storage in his study on hydrosol block-type melting cheeses. He reported that increasing the water content in these cheeses decreased external stickiness. According to the same researcher from another source, proteolysis caused by storage increased external stickiness. In our study, it was observed that the increase in the water ratio in the samples generally decreased the external adhesion. Cankurt [20], Yüksel [26], and Çavuş [23] are three researchers who worked on block-type melting cheese. In their studies and the studies of other researchers they cited, they reported that the external stickiness exhibited decreases and increases during storage and that this was due to the biochemical events occurring in the cheeses. 

Internal stickiness is defined by Tunick [58] as the value indicating the force applied to deform the cheese before breaking. It has been reported that the internal stickiness values of low-fat and full-fat Cheddar cheese decrease as ripening progresses during storage [59]. It was observed that the internal stickiness values of the block-type melting cheese we produced were between 0.79 and 0.86. When the internal stickiness values of the cheese samples were analyzed, it was found that they were between 0.82–0.85 on the first day, between 0.80–0.86 on the 15th day and between 0.79–0.85 on the 30th day. 

Internal stickiness (cohesiveness) is defined as the strength of the internal bonds between proteins and fats that provide the formation of the three-dimensional structure in cheese. It is reported that the change in the texture of cheeses during storage is due to the continuous breaking and re-establishment of protein bonds [20]. According to a researcher, the first day internal stickiness was found to be between 0.44–0.68 in caper cheese, 0.21–0.56 in transglutaminase cheese, and 0.79–0.83 in hydrosol and volatile oil cheese [20].

The crushing force required to make semi-solid cheese into a shape suitable for swallowing is called chewiness [60]. Chewability is not a direct characteristic of cheeses but is calculated by using the relationships between the values obtained from several parameters. The parameters used in the calculation of chewability are hardness, internal stickiness, and elasticity. The chewability value tells us the power required to chew the sample. The higher the chewability value of a sample, the more effort needed to chew that sample. Thus, in a way, the chewability value can actually be considered as non-chewability [20]. According to Cankurt [20], the chewability value decreases with increasing moisture content in the sample. He also reported that this value decreases with ripening. In other words, it is easier to chew cheeses with a high moisture content or matured cheeses. The chewiness values of the cheese samples to which we added carrot fiber and some gums in production followed a fluctuating course; these values are shown in Table 29. In the analyses, values of 329–2344 were determined. While the first day chewiness value was found to be 329–2344, it was found to be between 336 and 1706 in the 15th day analyses. In the 30th day analysis, it was found at between 403 and 1738. As can be seen, chewability values decreased over time in our samples. The important thing here is free water, because on the first day, it was observed that the chewability decreased as the water content increased in all samples but increased in the samples containing carrageenan due to the strong retention of water by carrageenan.

Gumminess values are calculated by taking the internal stickiness and hardness values of any food [61]. Gumminess is necessary to make a semi-solid food ready for swallowing. It is expressed as fragmentation force or number. It is defined numerically as the value obtained by multiplying hardness and internal stickiness [20]. As a result of the gumminess analysis of the block-type melting cheese we produced, it was determined that there were fluctuations in the values of the samples during storage. In the analyses, it was found that the gumminess values were between 776–6089; the lowest gumminess value was 776 on the 15th day analysis in the cheese sample to which 0.50% carrageenan gum was added, while the highest value was 6089 on the 30th day analysis in the control group cheese sample. Gumminess increased in all samples, but the increase was lower in the samples with high gum and fiber content as more water was added. Cankurt [20] reported that the gumminess of hydrosol and volatile oil samples and Yuksel [26] reported that the gumminess of egg block-type melted cheeses decreased fluctuatingly throughout storage. It is understood from this that the gums and carrot fiber we used prevented the gumminess values from decreasing. 

The rate at which the cheese returns to its original state after the initial compression is called the elasticity of the cheese [62]. In other words, it is expressed as the return of the cheese to its original state after the removal of the force applied on the cheese [63]. In the study in which it was used as a fat substitute in the production of kashkaval cheese, it was reported that the elasticity of the cheese samples produced fluctuated during the ripening period [21]. It was observed that the elasticity values of the cheese samples were between 0.41–0.46 on the first day. In the 15th day analyses, it was found to be between 0.38–0.49, and in the 30th day analyses, it was found to be between 0.38 and 0.50. As a result of the analysis of the elasticity values of the block-type melting cheese, it was observed that the first day elasticity value was between 0.27 and 0.62. In the 15th day analyses, it was between 0.30 and 0.57, and in the 30th day analyses, values between 0.27 and 0.60 were determined. According to Cankurt [62], the elasticity values of block-type melted cheese samples increased and then decreased. He found it to be between 0.39–0.45 on the first day and 0.42–0.48 at the end of 90 days. According to Yüksel [26], the elasticity values of egg block-type melted cheese samples decreased continuously. On the first day, the values were found to be between 0.46–0.54, and at the end of 60 days, between 0.38–0.50.

### 3.5. Conclusions and Some Suggestions Emerged from This Study

At the end of this study, it was understood that carrot fiber can be an alternative to other gums with its properties and yield aspect but that its usage rate should not be more than 2.5% in order not to cause sensory disturbance. Preventing carrot fiber from giving cheese an orange color and reducing the visibility of carrot fiber grains in cheese will increase sensory appeal. As a result, it is understood that carrot fiber can compete with other gums in terms of improving yield, texture, and sensory properties.

1. If carrot fibers are to be used in the production of block-type melting cheese, it is recommended to further reduce the size of the fibers.
2. It is recommended to keep carrot fiber between 2.5–5.0% and not to increase it further.
3. Although cheese yields are increased compared to the control, it is recommended that the amount of water added should not exceed 10%.
4. Although not included in the study, the storage of block-type melting cheeses was extended, and all cheeses were found to be more flavorful. Therefore, it is recommended that the shelf life of the cheeses produced in similar studies should be at least three months.
5. If the use of gum is preferred, it is recommended to prioritize Carrageenan, but if 0.5% is used, the amount of water added should be kept below 10%.
6. If the carrot fiber is to be produced by the researcher, it is recommended to store the fibers obtained in a place out of the light. Otherwise, the dulling and darkening of the fibers occurs.
7. Although there are studies on the use of other gums in dairy products, the use of carrot fiber is very limited. It is recommended that similar studies be carried out on the use of carrot fiber in products such as cream-type melting cheese and yoghurt.

## Figures and Tables

**Table 1:** Properties of Raw Milk Used in the Production of Melted Cheese.

Properties	Raw Cow Milk Values
pH	6.72
Total acidity (%La)	0.17
Dry matter (%)	12.30
Fat (%)	3.75
Fat Free Dry Matter (%)	8.55
Protein (%)	3.40

**Table 2:** Amounts of block-type melting cheese obtained from 100 L of milk (Kg).

Cheese Types	Quantity of Cheese Obtained (Kg)
Xanthan 0.25	10.78
Xanthan 0.50	11.38
Carrageenan 0.25	10.82
Carrageenan 0.50	11.38
Carrot fiber 2.5	11.25
Carrot fiber 5.0	12.12
Control	10.58

**Table 3:** Dry Matter Values (%) of Block-Type Melted Cheese Samples.

Cheese Types	Dry Matter
Xanthan 0.25	56.61 ± 0.15 ^b^
Xanthan 0.50	53.62 ± 0.14 ^d^
Carrageenan 0.25	56.30 ± 0.17 ^bc^
Carrageenan 0.50	53.87 ± 0.63 ^cd^
Carrot fiber 2.5	56.55 ± 0.41 ^b^
Carrot fiber 5.0	53.94 ± 0.91 ^cd^
Control	59.65 ± 1.22 ^a^

^a–d^: Small letters indicate differences between cheese samples (*p* < 0.05).

**Table 4:** Fat Values (%) of Block-Type Melted Cheese Samples.

Cheese Types	Fat Values
Xanthan 0.25	28.00 ± 0.71 ^b^
Xanthan 0.50	26.55 ± 0.07 ^c^
Carrageenan 0.25	27.50 ± 0.00 ^bc^
Carrageenan 0.50	26.25 ± 0.35 ^cd^
Carrot fiber 2.5	26.25 ± 0.35 ^cd^
Carrot fiber 5.0	25.00 ± 0.00 ^d^
Control	29.40 ± 0.28 ^a^

^a–d^: Small letters indicate differences between cheese samples (*p* < 0.05).

**Table 5:** Fat in Dry Matter Values (%) of Block-Type Melted Cheese Samples.

Cheese Types	Fat Values
Xanthan 0.25	49.46
Xanthan 0.50	49.52
Carrageenan 0.25	48.85
Carrageenan 0.50	48.73
Carrot fiber 2.5	46.42
Carrot fiber 5.0	46.35
Control	49.29

**Table 6:** Salt Values (%) of Block-Type Melting Cheese Samples.

Cheese Types	Salt Values
Xanthan 0.25	1.38 ± 0.01 ^bcd^
Xanthan 0.50	1.32 ± 0.03 ^cd^
Carrageenan 0.25	1.40 ± 0.03 ^b^
Carrageenan 0.50	1.35 ± 0.01 ^bcd^
Carrot fiber 2.5	1.39 ± 0.01 ^bc^
Carrot fiber 5.0	1.31 ± 0.02 ^d^
Control	1.48 ± 0.01 ^a^

^a–d^: Small letters indicate differences between cheese samples (*p* < 0.05).

**Table 7:** Salt Values in Dry Matter of Block-Type Melting Cheese Samples (%).

Cheese Types	Salt Values in Dry Matter
Xanthan 0.25	2.44
Xanthan 0.50	2.46
Carrageenan 0.25	2.49
Carrageenan 0.50	2.51
Carrot fiber 2.5	2.46
Carrot fiber 5.0	2.43
Control	2.48

**Table 8:** Protein Values of Block-Type Melted Cheese Samples (%).

Cheese Types	Protein Values
Xanthan 0.25	23.76 ± 0.34 ^ab^
Xanthan 0.50	22.29 ± 0.20 ^cd^
Carrageenan 0.25	23.03 ± 0.18 ^bcd^
Carrageenan 0.50	22.38 ± 0.21 ^cd^
Carrot fiber 2.5	23.15 ± 0.21 ^bc^
Carrot fiber 5.0	22.07 ± 0.20 ^d^
Control	24.48 ± 0.34 ^a^

^a–d^: Small letters indicate differences between cheese samples (*p* < 0.05).

**Table 9:** Ash Values (%) of Block-Type Melted Cheese Samples.

Cheese Types	Ash Values
Xanthan 0.25	3.70 ± 0.03 ^b^
Xanthan 0.50	3.62 ± 0.01 ^bc^
Carrageenan 0.25	3.67 ± 0.03 ^b^
Carrageenan 0.50	3.54 ± 0.01 ^cd^
Carrot fiber 2.5	3.68 ± 0.01 ^b^
Carrot fiber 5.0	3.52 ± 0.03 ^d^
Control	3.97 ± 0.01 ^a^

^a–d^: Small letters indicate differences between cheese samples (*p* < 0.05).

**Table 10:** pH Values of Block-Type Melted Cheese Samples.

Cheese Types		Storage Duration	
	Day 1	Day 15	Day 30
Xanthan 0.25	5.67 ± 0.01 ^Aa^	5.65 ± 0.01 ^Ab^	5.61 ± 0.01 ^Bbcd^
Xanthan 0.50	5.70 ± 0.01 ^Aa^	5.65 ± 0.01 ^Bb^	5.58 ± 0.01 ^Cd^
Carrageenan 0.25	5.67 ± 0.00 ^Ba^	5.70 ± 0.00 ^Aa^	5.65 ± 0.01 ^Cab^
Carrageenan 0.50	5.68 ± 0.01 ^Aa^	5.65 ± 0.00 ^Bb^	5.60 ± 0.00 ^Ccd^
Carrot fiber 2.5	5.68 ± 0.01 ^Aa^	5.67 ± 0.01 ^Ab^	5.64 ± 0.01 ^Aabc^
Carrot fiber 5.0	5.70 ± 0.01 ^Aa^	5.67 ± 0.01 ^Ab^	5.66 ± 0.01 ^Aa^
Control	5.63 ± 0.02 ^Aa^	5.66 ± 0.00 ^Ab^	5.64 ± 0.01 ^Aabc^

^a–d^: Lower case letters indicate differences between cheese samples, ^A–C^: Upper case letters indicate differences between storage days (*p* < 0.05).

**Table 11:** Total Acidity Values (%) of Block-Type Melted Cheese Samples.

Cheese Types		Storage Duration	
	Day 1	Day 15	Day 30
Xanthan 0.25	1.72 ± 0.01 ^Ba^	1.77 ± 0.01 ^ABa^	1.83 ± 0.04 ^Aa^
Xanthan 0.50	1.67 ± 0.01 ^Ba^	1.65 ± 0.01 ^Bb^	1.76 ± 0.00 ^Ab^
Carrageenan 0.25	1.69 ± 0.00 ^Aa^	1.64 ± 0.01 ^Cb^	1.67 ± 0.00 ^Bd^
Carrageenan 0.50	1.65 ± 0.07 ^Aa^	1.65 ± 0.02 ^Ab^	1.70 ± 0.01 ^Acd^
Carrot fiber 2.5	1.61 ± 0.04 ^Aab^	1.58 ± 0.01 ^Acd^	1.66 ± 0.01 ^Ad^
Carrot fiber 5.0	1.49 ± 0.00 ^Bbc^	1.55 ± 0.01 ^Ad^	1.54 ± 0.01 ^Ae^
Control	1.45 ± 0.01 ^Cc^	1.63 ± 0.00 ^Bbc^	1.75 ± 0.01 ^Abc^

^a–e^: Lower case letters indicate differences between cheese samples; ^A–C^: Upper case letters indicate differences between storage days (*p* < 0.05).

**Table 12:** L* Values of Block-Type Melted Cheese Samples.

Cheese Types		Storage Duration	
	Day 1	Day 15	Day 30
Xanthan 0.25	87.12 ± 0.26 ^Aa^	85.12 ± 0.29 ^Cd^	86.36 ± 0.18 ^Bc^
Xanthan 0.50	86.28 ± 0.15 ^ABb^	85.90 ± 0.30 ^Bc^	86.89 ± 0.33 ^Abc^
Carrageenan 0.25	87.10 ± 0.36 ^Aa^	86.77 ± 0.48 ^Ab^	87.46 ± 0.11 ^Aab^
Carrageenan 0.50	87.34 ± 0.15 ^Ba^	88.09 ± 0.18 ^Aa^	87.81 ± 0.26 ^ABa^
Carrot fiber 2.5	82.38 ± 0.16 ^Bc^	81.99 ± 0.17 ^Ce^	84.10 ± 0.05 ^Ad^
Carrot fiber 5.0	82.02 ± 0.11 ^Ac^	79.86 ± 0.15 ^Bf^	81.86 ± 0.40 ^Ae^
Control	87.41 ± 0.18 ^Aa^	86.24 ± 0.15 ^Bbc^	84.24 ± 0.09 ^Cd^

^a–f^: Lower case letters indicate differences between cheese samples; ^A–C^: Upper case letters indicate differences between storage days (*p* < 0.05).

**Table 13:** a* Values of Block-Type Melted Cheese Samples.

Cheese Types	Storage Duration		
	Day 1	Day 15	Day 30
Xanthan 0.25	3.15 ± 0.05 ^Bc^	2.94 ± 0.01 ^Cc^	3.49 ± 0.03 ^Acd^
Xanthan 0.50	2.97 ± 0.02 ^Bd^	2.53 ± 0.03 ^Cf^	3.56 ± 0.06 ^Ac^
Carrageenan 0.25	3.08 ± 0.03 ^Bc^	2.70 ± 0.04 ^Cd^	3.35 ± 0.02 ^Ae^
Carrageenan 0.50	2.92 ± 0.03 ^Bd^	2.88 ± 0.03 ^Bc^	3.39 ± 0.06 ^Ade^
Carrot fiber 2.5	4.90 ± 0.02 ^Bb^	5.00 ± 0.01 ^Ab^	5.00 ± 0.06 ^Ab^
Carrot fiber 5.0	5.49 ± 0.02 ^Ca^	5.86 ± 0.01 ^Ba^	6.20 ± 0.03 ^Aa^
Control	2.98 ± 0.03 ^Ad^	2.62 ± 0.01 ^Ce^	2.82 ± 0.01 ^Bf^

^a–f^: Lower case letters indicate differences between cheese samples; ^A–C^: Upper case letters indicate differences between storage days (*p* < 0.05).

**Table 14:** b* Values of Block-Type Melted Cheese Samples.

Cheese Types		Storage Duration	
	Day 1	Day 15	Day 30
Xanthan 0.25	29.99 ± 0.02 ^Cd^	30.66 ± 0.04 ^Bd^	31.02 ± 0.00 ^Af^
Xanthan 0.50	29.22 ± 0.09 ^Be^	29.07 ± 0.06 ^Be^	31.79 ± 0.03 ^Ad^
Carrageenan 0.25	30.47 ± 0.13 ^Cc^	30.86 ± 0.08 ^Bd^	32.24 ± 0.02 ^Ac^
Carrageenan 0.50	28.08 ± 0.03 ^Cf^	29.00 ± 0.11 ^Be^	31.44 ± 0.01 ^Ae^
Carrot fiber 2.5	32.56 ± 0.02 ^Cb^	33.53 ± 0.16 ^Bb^	35.17 ± 0.17 ^Ab^
Carrot fiber 5.0	33.99 ± 0.04 ^Ca^	34.70 ± 0.01 ^Ba^	37.37 ± 0.12 ^Aa^
Control	30.00 ± 0.02 ^Bd^	31.68 ± 0.05 ^Ac^	29.42 ± 0.03 ^Cg^

^a–g^: Lower case letters indicate differences between cheese samples; ^A–C^: Upper case letters indicate differences between storage days (*p* < 0.05).

**Table 15:** Fusibility Values (cm) of Block-Type Melting Cheese Samples.

Cheese Types		Storage Duration	
	Day 1	Day 15	Day 30
Xanthan 0.25	6.63 ± 0.18 ^Aab^	6.58 ± 0.00 ^Aabc^	6.50 ± 0.00 ^Aab^
Xanthan 0.50	6.04 ± 0.30 ^Abc^	5.91 ± 0.00 ^Ad^	6.17 ± 0.12 ^Ac^
Carrageenan 0.25	6.92 ± 0.12 ^Aa^	7.04 ± 0.18 ^Aa^	6.71 ± 0.06 ^Aa^
Carrageenan 0.50	6.17 ± 0.12 ^Abc^	6.08 ± 0.35 ^Acd^	6.21 ± 0.06 ^Abc^
Carrot fiber 2.5	6.21 ± 0.18 ^Bbc^	6.29 ± 0.06 ^Bbcd^	6.75 ± 0.00 ^Aa^
Carrot fiber 5.0	5.87 ± 0.06 ^Ac^	5.79 ± 0.06 ^Ad^	5.92 ± 0.12 ^Ac^
Control	5.95 ± 0.06 ^Bc^	6.87 ± 0.06 ^Aab^	6.79 ± 0.06 ^Aa^

^a–d^: Lower case letters indicate differences between cheese samples; ^A–B^: Upper case letters indicate differences between storage days (*p* < 0.05).

**Table 16:** TAMB Count of Block-Type Melted Cheese Samples (log cfu/g).

Cheese Types		Storage Duration	
	Day 1	Day 15	Day 30
Xanthan 0.25	5.42 ± 0.05 ^Cb^	5.89 ± 0.01 ^Bb^	6.08 ± 0.05 ^Ab^
Xanthan 0.50	5.80 ± 0.06 ^Ca^	6.11 ± 0.04 ^Ba^	6.32 ± 0.01 ^Aa^
Carrageenan 0.25	4.49 ± 0.01 ^Bd^	4.80 ± 0.05 ^Ac^	3.93 ± 0.04 ^Ce^
Carrageenan 0.50	4.74 ± 0.01 ^Bc^	5.80 ± 0.02 ^Ab^	5.79 ± 0.04 ^Ac^
Carrot fiber 2.5	3.75 ± 0.06 ^Bf^	4.00 ± 0.03 ^Ae^	2.87 ± 0.05 ^Cg^
Carrot fiber 5.0	3.15 ± 0.05 ^Bg^	3.33 ± 0.04 ^ABf^	3.51 ± 0.04 ^Af^
Control	4.02 ± 0.03 ^Be^	4.30 ± 0.01 ^Ad^	4.34 ± 0.10 ^Ad^

^a–g^: Lower case letters indicate differences between cheese samples; ^A–C^: Upper case letters indicate differences between storage days (*p* < 0.05).

**Table 17:** Total Yeast-Mold Count of Block-Type Melted Cheese Samples (log cfu/g).

Cheese Types		Storage Duration	
	Day 1	Day 15	Day 30
Xanthan 0.25	2.06 ± 0.03 ^Bbc^	2.88 ± 0.04 ^Ab^	2.93 ± 0.04 ^Ac^
Xanthan 0.50	2.32 ± 0.03 ^Babc^	3.19 ± 0.01 ^Aa^	3.24 ± 0.01 ^Aab^
Carrageenan 0.25	2.44 ± 0.06 ^Bab^	3.25 ± 0.02 ^Aa^	3.29 ± 0.01 ^Aab^
Carrageenan 0.50	2.65 ± 0.07 ^Ba^	3.21 ± 0.04 ^Aa^	3.35 ± 0.01 ^Aa^
Carrot fiber 2.5	2.15 ± 0.21 ^Bbc^	2.54 ± 0.08 ^ABc^	2.93 ± 0.04 ^Ac^
Carrot fiber 5.0	2.39 ± 0.13 ^Babc^	2.74 ± 0.06 ^ABb^	3.10 ± 0.08 ^Abc^
Control	2.00 ± 0.00 ^Bc^	2.30 ± 0.00 ^ABd^	2.39 ± 0.13 ^Ad^

^a–d^: Lower case letters indicate differences between cheese samples; ^A–B^: Upper case letters indicate differences between storage days (*p* < 0.05).

**Table 18:** TLAB Count of Block-Type Melted Cheese Samples (log cfu/g).

Cheese Types		Storage Duration	
	Day 1	Day 15	Day 30
Xanthan 0.25	5.13 ± 0.04 ^Cb^	5.73 ± 0.08 ^Bb^	6.38 ± 0.11 ^Ab^
Xanthan 0.50	5.61 ± 0.01 ^Ca^	6.07 ± 0.02 ^Ba^	6.90 ± 0.06 ^Aa^
Carrageenan 0.25	4.40 ± 0.00 ^Bd^	4.67 ± 0.08 ^Ac^	4.26 ± 0.04 ^Bc^
Carrageenan 0.50	4.63 ± 0.04 ^Cc^	5.68 ± 0.04 ^Bb^	6.75 ± 0.11 ^Aa^
Carrot fiber 2.5	3.53 ± 0.01 ^Bf^	3.84 ± 0.04 ^Ae^	2.48 ± 0.00 ^Ce^
Carrot fiber 5.0	2.90 ± 0.07 ^Bg^	3.13 ± 0.07 ^ABf^	3.36 ± 0.08 ^Ad^
Control	3.81 ± 0.02 ^Be^	4.09 ± 0.06 ^Ad^	4.18 ± 0.04 ^Ac^

^a–f^: Lower case letters indicate the difference between cheese samples, ^A–C^: Upper case letters indicate the difference between storage days (*p* < 0.05).

**Table 19:** Coliform Group Bacteria Count of Block-Type Melted Cheese Samples (log cfu/g).

Cheese Types	St	Storage Duration	
	Day 1	Day 15	Day 30
Xanthan 0.25	1.59 ± 0.16 ^c^	***	***
Xanthan 0.50	2.82 ± 0.05 ^a^	***	***
Carrageenan 0.25	1.00 ± 0.00 ^d^	***	***
Carrageenan 0.50	1.98 ± 0.04 ^b^	***	***
Carrot fiber 2.5	***	***	***
Carrot fiber 5.0	***	***	***
Control	***	***	***

^a–d^: Lower case letters indicate differences between cheese samples (*p* < 0.05). ***: Not detected.

**Table 20:** Appearance Values of Block-Type Melted Cheese.

Cheese Types	Storage Duration		
	Day 1	Day 15	Day 30
Xanthan 0.25	7.3 ± 1.25 ^Aa^	7.5 ± 1.43 ^Aa^	7.9 ± 0.88 ^Aab^
Xanthan 0.50	7.0 ± 1.70 ^Aa^	7.6 ± 1.17 ^Aa^	7.7 ± 1.06 ^Aab^
Carrageenan 0.25	7.3 ± 1.49 ^Aa^	7.5 ± 0.71 ^Aa^	7.8 ± 1.03 ^Aab^
Carrageenan 0.50	7.2 ± 1.55 ^Aba^	6.7 ± 1.25 ^Ba^	8.1 ± 0.88 ^Aab^
Carrot fiber 2.5	7.2 ± 1.75 ^Aa^	7.1 ± 1.73 ^Aa^	7.2 ± 1.55 ^Aab^
Carrot fiber 5.0	6.9 ± 1.73 ^Aa^	6.7 ± 1.34 ^Aa^	6.6 ± 1.43 ^Ab^
Control	7.5 ± 1.43 ^Aa^	8.0 ± 0.82 ^Aa^	8.5 ± 0.70 ^Aa^

^a–b^: Lower case letters indicate differences between cheese samples; ^A–B^: Upper case letters indicate differences between storage days (*p* < 0.05).

**Table 21:** Texture Values of Block-Type Melted Cheese.

Cheese Types	Storage Duration		
	Day 1	Day 15	Day 30
Xanthan 0.25	7.4 ± 0.84 ^Aa^	7.8 ± 1.23 ^Aa^	8.0 ± 0.82 ^Aab^
Xanthan 0.50	5.5 ± 1.43 ^Ba^	6.5 ± 1.27 ^ABa^	7.5 ± 1.18 ^Aab^
Carrageenan 0.25	5.9 ± 1.20 ^Ba^	6.5 ± 0.97 ^Ba^	7.7 ± 1.06 ^Aab^
Carrageenan 0.50	5.7 ± 1.16 ^Ba^	6.8 ± 1.75 ^ABa^	8.0 ± 1.05 ^Aab^
Carrot fiber 2.5	7.1 ± 1.73 ^Aa^	6.9 ± 1.73 ^Aa^	6.9 ± 1.66 ^Ab^
Carrot fiber 5.0	6.8 ± 1.81 ^Aa^	6.8 ± 1.55 ^Aa^	6.6 ± 1.35 ^Ab^
Control	6.9 ± 1.73 ^Ba^	8.1 ± 0.99 ^ABa^	8.6 ± 0.70 ^Aa^

^a–b^: Lower case letters indicate differences between cheese samples; ^A–B^: Upper case letters indicate differences between storage days (*p* < 0.05).

**Table 22:** Taste Values of Block-Type Melted Cheese.

Cheese Types	Storage Duration		
	Day 1	Day 15	Day 30
Xanthan 0.25	7.0 ± 0.94 ^Bab^	7.5 ± 1.43 ^ABab^	8.4 ± 0.84 ^Aa^
Xanthan 0.50	6.1 ± 0.99 ^Aab^	6.9 ± 1.45 ^Aab^	7.5 ± 1.43 ^Aab^
Carrageenan 0.25	6.8 ± 1.32 ^Aab^	7.0 ± 1.49 ^Aab^	7.8 ± 1.40 ^Aab^
Carrageenan 0.50	5.9 ± 1.20 ^Bb^	6.6 ± 1.35 ^ABab^	7.9 ± 1.45 ^Aa^
Carrot fiber 2.5	7.0 ± 1.63 ^Aab^	6.4 ± 2.12 ^Aab^	6.3 ± 2.63 ^Aab^
Carrot fiber 5.0	5.7 ± 1.89 ^Ab^	5.6 ± 1.78 ^Ab^	5.6 ± 2.32 ^Ab^
Control	7.8 ± 1.23 ^Aa^	8.2 ± 0.63 ^Aa^	8.4 ± 0.84 ^Aa^

^a–b^: Lower case letters indicate differences between cheese samples; ^A–B^: Upper case letters indicate differences between storage days (*p* < 0.05).

**Table 23:** All Impression Values of Block-Type Melted Cheeses.

Cheese Types	Storage Duration		
	Day 1	Day 15	Day 30
Xanthan 0.25	7.1 ± 0.99 ^Aa^	7.8 ± 1.14 ^Aa^	8.0 ± 0.82 ^Aa^
Xanthan 0.50	5.8 ± 1.23 ^Ba^	6.9 ± 1.45 ^ABa^	7.6 ± 1.17 ^Aab^
Carrageenan 0.25	6.8 ± 1.55 ^Aa^	7.2 ± 1.23 ^Aa^	7.8 ± 1.14 ^Aab^
Carrageenan 0.50	5.9 ± 1.10 ^Ba^	6.3 ± 2.26 ^ABa^	7.9 ± 1.20 ^Aa^
Carrot fiber 2.5	7.0 ± 1.76 ^Aa^	7.1 ± 1.45 ^Aa^	7.0 ± 1.76 ^Aab^
Carrot fiber 5.0	5.9 ± 1.79 ^Aa^	6.0 ± 1.63 ^Aa^	6.1 ± 1.79 ^Ab^
Control	7.6 ± 1.17 ^Aa^	7.9 ± 1.45 ^Aa^	8.5 ± 0.85 ^Aa^

^a–b^: Lower case letters indicate differences between cheese samples; ^A–B^: Upper case letters indicate differences between storage days (*p* < 0.05).

**Table 24:** Hardness (g) Values of Block-Type Melting Cheeses.

Cheese Types		Storage Duration	
	Day 1	Day 15	Day 30
Xanthan 0.25	2259 ± 12.73 ^Bb^	1658 ± 25.46 ^Ce^	3922 ± 2.83 ^Ac^
Xanthan 0.50	1189 ± 12.73 ^Bf^	1829 ± 12.73 ^Ad^	1206 ± 8.49 ^Bf^
Carrageenan 0.25	1847 ± 9.90 ^Bd^	1795 ± 21.21 ^Bd^	2281 ± 7.07 ^Ae^
Carrageenan 0.50	1710 ± 14.14 ^Be^	974 ± 19.80 ^Cf^	2278 ± 11.31 ^Ae^
Carrot fiber 2.5	2261 ± 26.87 ^Cb^	2784 ± 5.66 ^Bb^	5057 ± 24.04 ^Ab^
Carrot fiber 5.0	2139 ± 26.87 ^Cc^	2274 ± 19.80 ^Bc^	2565 ± 7.07 ^Ad^
Control	4035 ± 12.73 ^Ba^	3487 ± 9.90 ^Ca^	7139 ± 12.73 ^Aa^

^a–f^: Lower case letters indicate differences between cheese samples; ^A–C^: Upper case letters indicate differences between storage days (*p* < 0.05).

**Table 25:** External Stickiness Values of Block-Type Melting Cheeses.

Cheese Types		Storage Duration	
	Day 1	Day 15	Day 30
Xanthan 0.25	−250 ± 14.14 ^Cd^	−85 ± 7.07 ^Cb^	−159 ± 8.49 ^Bc^
Xanthan 0.50	−172 ± 5.66 ^Bc^	−378 ± 5.66 ^Ce^	−24 ± 1.41 ^Aa^
Carrageenan 0.25	−316 ± 8.49 ^Be^	−108 ± 4.24 ^Ab^	−458 ± 11.31 ^Cf^
Carrageenan 0.50	−760 ± 14.14 ^Bf^	−103 ± 9.90 ^Ab^	−118 ± 9.90 ^Ab^
Carrot fiber 2.5	−131 ± 4.24 ^Ab^	−242 ± 4.24 ^Bd^	−385 ± 7.07 ^Ce^
Carrot fiber 5.0	−132 ± 5.66 ^Bb^	−211 ± 1.41 ^Cc^	−35 ± 7.07 ^Aa^
Control	−76 ± 2.83 ^Ba^	−57 ± 4.24 ^Aa^	−313 ± 4.24 ^Cd^

^a–f^: Lower case letters indicate differences between cheese samples; ^A–C^: Upper case letters indicate differences between storage days (*p* < 0.05).

**Table 26:** Elasticity Values of Block-Type Melting Cheeses.

Cheese Types		Storage Duration	
	Day 1	Day 15	Day 30
Xanthan 0.25	0.42 ± 0.03 ^Acd^	0.30 ± 0.03 ^Ac^	0.36 ± 0.06 ^Abc^
Xanthan 0.50	0.34 ± 0.03 ^Ad^	0.39 ± 0.01 ^Abc^	0.42 ± 0.03 ^Aabc^
Carrageenan 0.25	0.69 ± 0.04 ^Ab^	0.55 ± 0.07 ^Aab^	0.60 ± 0.07 ^Aa^
Carrageenan 0.50	0.62 ± 0.01 ^Aa^	0.43 ± 0.01 ^Babc^	0.27 ± 0.10 ^Bc^
Carrot fiber 2.5	0.63 ± 0.03 ^Ab^	0.54 ± 0.06 ^ABab^	0.38 ± 0.04 ^Babc^
Carrot fiber 5.0	0.31 ± 0.01 ^Cd^	0.44 ± 0.03 ^Babc^	0.57 ± 0.01 ^Aab^
Control	0.47 ± 0.04 ^ABc^	0.57 ± 0.06 ^Aa^	0.29 ± 0.03 ^Bc^

^a–d^: Lower case letters indicate differences between cheese samples; ^A–C^: Upper case letters indicate differences between storage days (*p* < 0.05).

**Table 27:** Internal Stickiness Values of Block-Type Melting Cheeses.

Cheese Types		Storage Duration	
	Day 1	Day 15	Day 30
Xanthan 0.25	0.82 ± 0.03 ^Aa^	0.83 ± 0.01 ^Aa^	0.85 ± 0.03 ^Aa^
Xanthan 0.50	0.82 ± 0.03 ^Aa^	0.81 ± 0.01 ^Aa^	0.79 ± 0.07 ^Aa^
Carrageenan 0.25	0.83 ± 0.03 ^Aa^	0.81 ± 0.01 ^Aa^	0.85 ± 0.07 ^Aa^
Carrageenan 0.50	0.85 ± 0.04 ^Aa^	0.80 ± 0.07 ^Aa^	0.82 ± 0.04 ^Aa^
Carrot fiber 2.5	0.82 ± 0.03 ^Aa^	0.82 ± 0.06 ^Aa^	0.84 ± 0.03 ^Aa^
Carrot fiber 5.0	0.83 ± 0.06 ^Aa^	0.82 ± 0.06 ^Aa^	0.83 ± 0.04 ^Aa^
Control	0.82 ± 0.06 ^Aa^	0.86 ± 0.06 ^Aa^	0.85 ± 0.01 ^Aa^

^a^: Lower case letters indicate differences between cheese samples; ^A^: Upper case letters indicate differences between storage days (*p* < 0.05).

**Table 28:** Gumminess Values of Block-Type Melted Cheese Samples.

Cheese Types		Storage Duration	
	Day 1	Day 15	Day 30
Xanthan 0.25	1847 ± 4.24 ^Bb^	1376 ± 8.49 ^Cf^	3348 ± 4.24 ^Ac^
Xanthan 0.50	970 ± 7.07 ^Bf^	1482 ± 2.83 ^Ad^	952 ± 2.83 ^Bg^
Carrageenan 0.25	1523 ± 4.24 ^Bd^	1446 ± 4.24 ^Ce^	1943 ± 8.49 ^Ae^
Carrageenan 0.50	1446 ± 2.83 ^Be^	776 ± 5.66 ^Cg^	1858 ± 11.31 ^Af^
Carrot fiber 2.5	1850 ± 4.24 ^Cb^	2284 ± 5.66 ^Bb^	4239 ± 5.66 ^Ab^
Carrot fiber 5.0	1782 ± 4.24 ^Cc^	1873 ± 4.24 ^Bc^	2116 ± 8.49 ^Ad^
Control	3308 ± 11.31 ^Ba^	2999 ± 1.41 ^Ca^	6089 ± 12.72 ^Aa^

^a–f^: Lower case letters indicate differences between cheese samples; ^A–C^: Upper case letters indicate differences between storage days (*p* < 0.05).

**Table 29:** Chewiness Values of Block-Type Melted Cheese Samples.

Cheese Types		Storage Duration	
	Day 1	Day 15	Day 30
Xanthan 0.25	769 ± 2.83 ^Be^	415 ± 7.07 ^Cf^	1206 ± 8.49 ^Ac^
Xanthan 0.50	329 ± 1.41 ^Cg^	583 ± 4.24 ^Ae^	403 ± 4.24 ^Bf^
Carrageenan 0.25	1050 ± 4.24 ^Bd^	795 ± 2.83 ^Cd^	1157 ± 9.90 ^Ad^
Carrageenan 0.50	2344 ± 4.24 ^Aa^	336 ± 8.49 ^Cg^	501 ± 5.66 ^Be^
Carrot fiber 2.5	1158 ± 11.31 ^Cc^	1228 ± 4.24 ^Bb^	1603 ± 4.24 ^Ab^
Carrot fiber 5.0	555 ± 7.07 ^Cf^	820 ± 1.41 ^Bc^	1207 ± 5.66 ^Ac^
Control	1541 ± 1.41 ^Cb^	1706 ± 4.24 ^Ba^	1738 ± 7.07 ^Aa^

^a–g^: Lower case letters indicate differences between cheese samples; ^A–C^: Upper case letters indicate differences between storage days (*p* < 0.05).

## Data Availability

The original contributions presented in the study are included in the article, further inquiries can be directed to the corresponding author.
